# Oxidative Stress in Mussel *Mytilus trossulus* Induced by Different-Sized Plastics

**DOI:** 10.3390/jox14040097

**Published:** 2024-11-22

**Authors:** Nadezhda Vladimirovna Dovzhenko, Valentina Vladimirovna Slobodskova, Andrey Alexandrovich Mazur, Sergey Petrovich Kukla, Aleksandra Anatolyevna Istomina, Victor Pavlovich Chelomin, Dmitriy Denisovich Beskhmelnov

**Affiliations:** Il’ichev Pacific Oceanological Institute, Far Eastern Branch, Russian Academy of Sciences, 690041 Vladivostok, Russia

**Keywords:** polyethylene, polystyrene, lysosome membrane stability, DNA damage, ecotoxicology, antiradical activity

## Abstract

Polyethylene and polystyrene are massively used around the world in various applications and are the most abundant plastic waste. Once in the marine environment, under the influence of physical and chemical factors, plastic products degrade, changing from the size category of macroplastics to microplastics. In order to study the effect of plastic on marine organisms, we modeled the conditions of environmental pollution with different-sized plastic—polystyrene microparticles of 0.9 µm and macro-sized polyethylene fragments of 10 cm—and compared their effect on biochemical parameters in the tissues of the bivalve mollusk *Mytilus trossulus*. Using biomarkers, it was found that regardless of the size and type of polymer, polystyrene microparticles and polyethylene macrofragments induced the development of oxidative stress in mussels. A significant decrease in the level of lysosomal stability in mussel hemocytes was observed. Increases in the level of DNA damage and the concentration of malonic dialdehyde in the cells of gills and the digestive gland were also shown. The level of total antiradical activity in cells varied and had a tissue-specific character. It was shown that both ingested polystyrene particles and leachable chemical compounds from polyethylene are toxic for mussels.

## 1. Introduction

Plastic fragments and microparticles account for about 60–90% of ocean debris on the planet, significantly affecting the quality of the aquatic environment and the hydrobionts that inhabit it, and plastic entering the environment is becoming an integral component of global pollution [1,2]. Due to ocean currents, plastics have spread to all regions of the world’s oceans, forming large-scale debris accumulations and migrating at the surface and in the water column [2]. Another problem of plastic debris in the marine environment is its constant defragmentation. Under the influence of ultraviolet light, friction, and other physical and chemical factors, plastic products break down into smaller fragments, resulting in the formation of micro- and nanoparticles [3]. To date, the level of microparticles in water in littoral zones and open sea waters of different regions of the world ranges from 0.001 to 140 particles/m^3^, and in bottom sediments, from 0.2 to 8766 particles/m^3^ [4]. Therefore, addressing the problem of plastic pollution due to its ubiquity in the ocean is considered one of the most important challenges today [5,6].

In the presence of plastic waste, aquatic organisms are negatively exposed both physically and chemically. Fragments of plastic objects can damage the cover of hydrobionts, be mistaken for food, and mechanically restrict movements [4]. By penetrating into marine organisms, plastic can damage the mucous membranes of organs and tissues, as well as accumulate in the gastrointestinal tract. This can result in serious damage to biochemical and physiological processes [7,8,9,10]. The presence of plastic in the environment also poses a threat of chemical exposure, as its production is based on mixing a base polymer with various additive components that impart certain properties to the final plastic product in order to reach performance standards [11]. According to various estimates, polymers include from 300 to 400 constituent components, among which there are hazardous monomers, low-molecular-weight oligomer fragments, synthetic stabilizers, catalysts, and various chemical additives, most of which are not covalently bonded to the polymer, dyes containing metal ions, plasticizers, phthalates, bisphenol-a, polychlorinated biphenyls, etc. [12,13]. Through interaction with the environment, these additives can actively leach into the water and become bioavailable to living organisms. Because of interaction with the environment, these additives actively leached into the water. Through the toxic effects of leaching chemical constituents into water, among which the most toxic are hydrophobic organic compounds and metals, the bioavailability of plastic to living organisms is observed. Leached chemical compounds trigger mechanisms of oxyradical generation in cells, leading to the development of oxidative stress, DNA molecule damage, and irreversible pathological processes, posing a life threat to aquatic organisms [14,15,16]. The degree of leaching of additives from polymers and leachate composition is influenced by the properties of compounds, the degree of amorphous or crystalline structure of the polymer, and the persistence of chemical bonds between additives and the polymer [17].

There are now several studies on the toxicity of individual additives migrating from plastic materials into the water, causing acute toxicity in hydrobionts [16,18,19]. It has been shown in different studies that marine organisms are prone to ingest plastic particles [20,21], but very few studies have investigated the toxicity of polymers to hydrobionts surrounded by plastic fragments not consumed by them [22].

In addition to releasing toxic substances into the environment, multi-sized plastics, mainly micro- and nanoplastics, actively penetrate trophic chains since marine inhabitants feeding on plastic particles mistake them for food objects [21,23]. Swallowed plastic particles damage internal organs; have a negative impact on the nervous, reproductive, and immune systems; and stop cell growth and reproduction by releasing chemicals inside the body [24,25,26]. At the biochemical level, active generation of free radicals and changes in metabolic processes are observed in cells. Thus, polymers pose a serious threat to marine organisms, being a multiple stressor due to their negative properties. Various studies at the molecular level have shown that plastics disrupt the redox balance in cells of living organisms, described in the literature as oxidative stress [27,28,29]. Molecular markers (biomarkers) of oxidative stress—the earliest indicators of environmental pollution—are used to diagnose such changes at different stages. As a rule, the use of these markers in ecotoxicological monitoring assumes a reliable correlation between the activity of metabolic processes and the level of environmental pollution [13,29,30].

Fragments of products made from polyethylene and polystyrene are the most common in plastic pollution both on land and in the ocean. These polymers are massively used around the world in applications ranging from light industry to medicine [31,32]. Moreover, the most commonly used items made from these types of plastic are disposable items [2,6]. In recent decades, the level of plastic waste pollution, dominated by polystyrene and polyethylene, has been increasing in the coastal zones of the Asia–Pacific region due to the increase in population density in the region and the associated increasing of economic activity in the region [33,34]. In order to understand the effects of this pollution, it is necessary to study the responses of hydrobionts to different types of plastics and their additives that are leached into the marine environment.

Therefore, the aim of this study was to evaluate the effects of polyethylene macrofragments and polystyrene microparticles on the Pacific mussel *Mytilus trossulus* and to carry out a comparative assessment of the toxicity of certain types of plastic on filter-feeding mollusks. To evaluate the toxic effect of polymers on living organisms, markers of oxidative stress were used: stability of lysosome membranes, DNA molecule damage, changes in the level of lipid peroxidation products, and integrated assessment of the level of antioxidant system activity.

## 2. Materials and Methods

### 2.1. Site of Bivalves Collection

Adults *M. trossulus* of equal age and size (6.1 ± 0.9 cm) were collected in the marine experimental station “Popov Island” of the Alekseev Bay in the Sea of Japan (42°59′ N; 131°43′ E). Mollusks were acclimated for 168 h in laboratory conditions in glass tanks in natural seawater. The water temperature in the tanks was +16 °C and salinity was 32.6‰. Water and air temperature were recorded every 12 h. During the period of acclimation and experiment, the mollusks where not fed.

### 2.2. Description of the Experiments

Two experiments were carried out. Experiment 1 (with HDPE fragments) was conducted for 72 h in two tanks—“control” (without exposure) and “experiment”. The volume of water in each tank was 25 L. There was 0.5 L of water for each mollusk. HDPE fragments were placed in the experimental tank with mussels. As HDPE fragments in the work, we used the film (produced in Russia, State Standard for HDPE film (16338-85)). The area of one fragment was 0.01 m^2^, the total area of fragments per experimental tank was 1 m^2^ (0.04 m^2^/L). The aeration of water in the tanks was carried out with the help of compressors, which, at the same time, created intensive wave flows of water, contributing to the washing out of intrinsic organic substances of the polyethylene film. During the experiment the filtration system was switched off, the water in the tanks with HDPE fragments and control did not change.

Experiment 2 (with polystyrene microspheres—µPS) lasted 72 h. Mussels were placed in the “control” (without exposure) and “experimental” tanks, with the rate of 0.5 L of water per one mussel. The volume of water in each tank was 25 L. The water and air temperature during the experiment was stable (+16 °C). Water and air temperatures were recorded every 12 h. µPS solution was added to the experimental tank with mollusks at a concentration of 10^5^ pcs/L. A standard solution of µPS (Cat. №6-1-0090, Tianjin BaseLine ChromTech Research Centre, China) was used to prepare a working solution of “microplastic”. The diameter of the microspheres was 0.9 μm. Water aeration in the tanks was carried out using compressors, which simultaneously created intense wave water flows, allowing the microspheres to remain in the water column and not settle to the bottom of the tank, as well as washing out their own persistent organic matter. Water changes in the control tank and with µPS were performed every 24 h. During the experiment, the filtration system was switched off; the water in the tanks with microspheres PS and control was not changed.

No mortality of *M. trossulus* was observed during both experiments. At the end of the exposure time in both experiments, 20 *M. trossulus* individuals from the control and experimental groups were used to determine the stability of the lysosomal membrane, 15 were used to determine % DNA damage, and 10 were used to prepare homogenates for the determination of MDA content and an index of antiradical activity.

All procedures in the present work, as well as the mollusks disposal methods, were approved by the Commission on Bioethics at the V.I. Il’ichev Pacific Oceanological Institute, Far Eastern Branch of Russian Academy of Science (protocol №16 and date of approval 15 April 2021), Vladivostok, Russia.

### 2.3. Cytochemical Methods

The level of DNA damage was determined using the alkaline comet assay, which is adapted to marine organisms [35]. Mollusks gills and the digestive gland were removed and gently cut in isotonic solution; then, gel slides were prepared (an amount of 50 µL of cell suspension was mixed with low gelling temperature agarose (100 µL), cell–agarose suspension was stirred and transferred to a slide containing normal gelling temperature agarose and covered with a coverslip), and the slide was then incubated in the lysis solution for 1 h in a light-protected place at 4 °C. After this, the slides were incubated in electrophoresis buffer (pH > 13) for 40 min. This was followed by electrophoresis for 15 min. After neutralization, the slides were stained with SYBR Green. The DNA comets were visualized and registered using a scanning fluorescence microscope (Carl Zeiss, AxioImager A1) equipped with a digital camera AxioCam MRc. The mollusks in the control and experimental groups were analyzed with 15 slides per group (1 slide = 1 mussel). Each slide contained no less than 50 comets. The program Casp 1.2.2 software (CASPlab, Wroclaw, Poland) was used to process digital images.

Lysosome membrane stability (LMS) in mollusk hemolymph was assessed using a cytochemical method based on the capture of a dye by lysosomes, the retention time of which shows the degree of damage to the membranes of the organelle [36]. Hemolymph from the mollusk closure muscle was sampled using a 1 mL hypodermic syringe. A total of 0.1 mL of hemolymph was taken from each individual. The incubation time of samples with the dye was 15 min. The stained samples were viewed under a microscope for 90 min at 15 min intervals.

### 2.4. Biochemical Methods

For determination of integral antiradical activity (IAA) and malonic dialdehyde (MDA) content, digestive gland and gill tissues were homogenized in chilled phosphate buffer (+4 °C) at a ratio of 1:10 g/mL (0.1 M, pH 7.0). Homogenates were centrifuged at 10,000 rpm for 40 min at +4 °C. Integral antiradical activity in tissue supernatant was determined by a method based on the ability of the antioxidant system of cells to reduce the radical cation ABTS+ [37]. Determination of the content of lipid peroxidation product-malonic dialdehyde in cells was carried out according to the colorimetric method [38].

### 2.5. Statistical Analysis

Statistical processing of the obtained results was performed using statistical tools MS Office Excel and Statistica 10 (StatSoft, Tulsa, OK, USA) program package: the arithmetic mean and standard deviation were calculated. The assumptions of normality and homogeneity were assessed using the Levene and Shapiro–Wilk tests, respectively. Normality was not observed, and nonparametric Mann–Whitney U-tests were performed. Differences were considered statistically significant at *p* < 0.05.

## 3. Results

**Figure 1:**
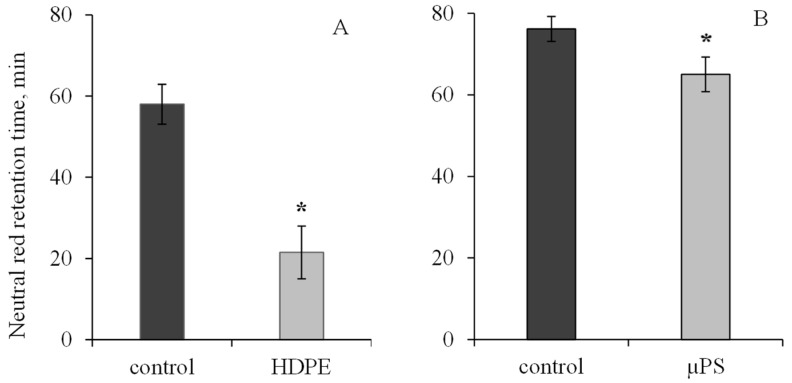
Changes in the lysosome membranes stability in *M. trossulus* hemolymph under the influence of different types of plastic ((**A**) high-density polyethylene fragments; (**B**) polystyrene microspheres) (mean ± standard deviation; *n* = 20). * Difference from control is significant at *p* < 0.05.

During short-term exposure of *M. trossulus* to polymers of different types and sizes, we revealed visible changes in biochemical parameters in mollusk tissues, indicating the development of oxidative stress and destructive processes in the cellular apparatus of the cell. Exposure of mollusks to polyethylene resulted in a 2.7-fold decrease in lysosome membrane stability (LMS) of hemocytes relative to the control. In the control group, the ability of lysosome membrane to retain dye was 58 ± 4.91 min, and under PE exposure, 21.5 ± 6.5 min. In the presence of µPS in mollusk hemocytes, the NR dye retention time was also found to decrease to 65 ± 4.24 min in comparison with the control (76.14 ± 3.05 min). (Figure 1).

**Figure 2:**
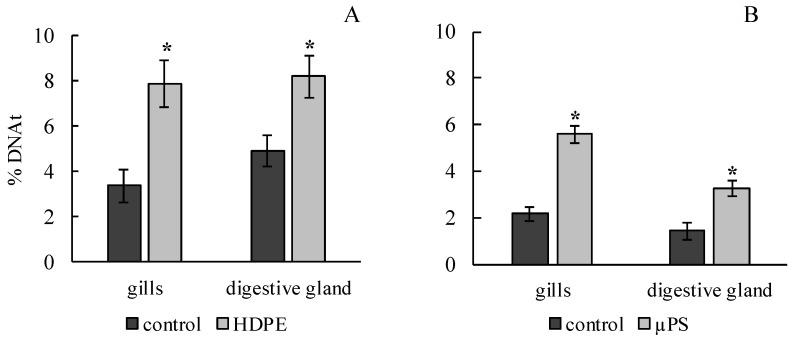
Damage of DNA molecule in cells of gills and digestive gland of *M. trossulus* under the influence of different types of plastic ((**A**) high-density polyethylene fragments; (**B**) polystyrene microspheres) (mean ± standard deviation; *n* = 15). * Difference from control is significant at *p* < 0.05.

The results of the comet assay showed that the effects of both polyethylene and polysterol caused a significant increase in the DNA damage in the gills and digestive gland of *M. trossulus*. In the experiment on exposure to polyethylene, it was 3.4 ± 0.7 and 4.95 ± 0.68% of DNA in the tail, compared to 7.89 ± 1.01 and 8.23 ± 0.93 in the control group for the gills and digestive gland, respectively. In the presence of µPS, these values were 5.6 ± 0.36 and 3.29 ± 0.35 compared to 2.17 ± 032 and 1.43 ± 0.34% of DNA in the tail for the gills and digestive gland, respectively (Figure 2).

**Figure 3:**
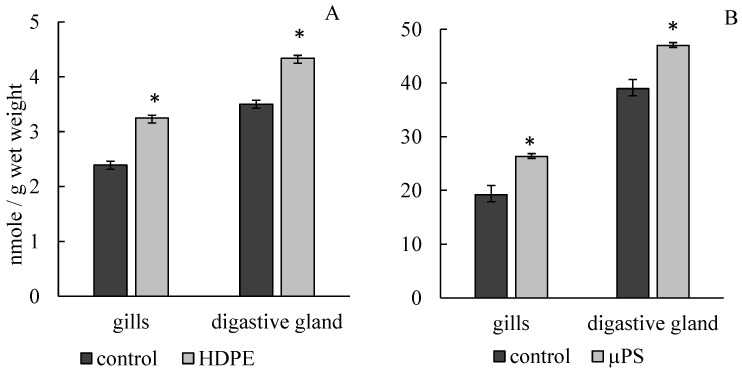
Changes in malonic dialdehyde content in cells of gills and digestive gland of *M. trossulus* under the influence of different types of plastic ((**A**) high-density polyethylene fragments; (**B**) polystyrene microspheres) (mean ± standard deviation; *n* = 10). * Difference from control is significant at *p* < 0.05.

The presence of PE fragments and PS microparticles in mussel tanks caused the development of lipid peroxidation processes, which led to a significant increase in MDA concentration in gill and digestive gland cells. Under the influence of PE, the MDA concentration in gills increased to 3.25 ± 0.05 mmol/g wet weight compared to the control (2.39 ± 0.09 mmol/g wet weight). In the presence of µPS in water, MDA level in gill cells also increased to 26.34 ± 2.22 mmol/g wet weight compared to control 19.2 ± 1.32 mmol/g wet weight. In digestive gland cells under PE exposure, MDA level increased to 4.34 ± 0.09 mmol/g wet weight compared to control 3.5 ± 0.073 mmol/g wet weight, with µPS increased to 47.01 ± 1.97 mmol/g wet weight against control 38.94 ± 1.69 mmol/g wet weight (Figure 3).

**Figure 4:**
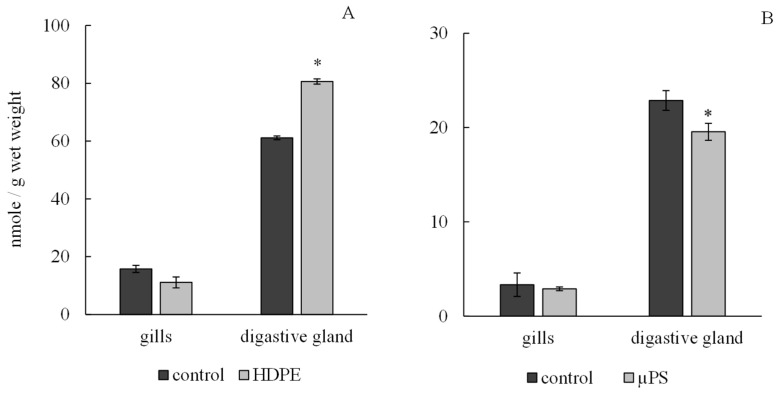
Changes in integral antiradical activity in cells of gills and digestive gland of *M. trossulus* under the influence of different types of plastic ((**A**) high-density polyethylene fragments; (**B**) polystyrene microspheres) (mean ± standard deviation; *n* = 10) * Difference from control is significant at *p* < 0.05.

According to the results, the presence of plastic in tanks caused a tissue-specific reaction of the antioxidant system of mollusks. Under exposure to polyethylene and µPS, the level of integrated antiradical activity (IAA) in the gills of mollusks did not change significantly and was 11.11 ± 0.65 nmol/g wet weight 2.9 ± 0.09 nmol/g wet weight for polyethylene and µPS fragments and at control values of 15.76 ± 0.91 nmol/g wet weight and 3.34 ± 1.05 nmol/g wet weight, respectively, while in digestive gland cells, there was a significant decrease in IAA under the influence of µPS from control values of 22.87 ± 1.24 nmol/g wet weight to 19.55 ± 0.2 nmol/g wet weight. However, the effect of polyethylene caused an increase in the IAA level in digestive gland cells to 80.61 ± 1.22 nmol/g wet weight compared to control 61.13 ± 1.9 nmol/g wet weight (Figure 4).

## 4. Discussion

In this work, a short-term experimental model of 72 h was used, assuming that under these conditions, the time would be sufficient to leach some concentration of chemical additives in PE and PS [13,39,40,41]. Despite the short time period, the results obtained revealed significant changes in biochemical parameters in mussel tissues.

In Experiment 1, the mussels had no direct contact with HDPE. All polymer fragments were on the surface and in the water column, while the mollusks were distributed on the bottom of the aquarium and attached to it by byssus threads. In earlier studies on *Daphnia magna*, it was shown that the leaching of additives from polymers, particularly HDPE, occurs in a very short time (48 h) [12,42]. At the outcome of our short-term experiment (72 h), biomarkers indicated the toxic effects of the leached substances from HDPE and the active development of oxidative stress in mollusk tissues, which was indicated by an increase in MDA content and a differential antioxidant system response (IAA) in mollusk tissues. The results of our studies also showed the bioavailability and toxicity of short-term effects of leachable substances from HDPE fragments on the lysosomal apparatus of mussels; in the hemolymph of experimental mollusks, the LMS decreased two-fold. Some authors have shown that a complex mixture of additives is leached from polymers into water, accumulates in the organism, and causes various processes such as lysosomal disorders, DNA molecule destruction, and, as a consequence, the development of oxidative stress [11,43,44,45]. In addition, studies have clearly demonstrated that the greatest toxic effect on *Mytilus galloprovincialis* mollusks is exerted by leached hydrophobic organic compounds, which activate the process of generating reactive oxygen species [45].

Microplastic particles also release endogenous substances both into the water and within the living organism. Therefore, in this case, the toxic effect of microplastics would be achieved by both mechanical and chemical damage. In the experiment with polystyrene, microparticles actively interacted with mussels by penetrating inside the mollusk with water. In our work, we used PS microspheres with a diameter of 0.9 μm, considering that particles of this size would be absorbed by mussels on par with their usual food. In bivalves, microplastic penetration has several possible pathways. Microplastics, in interaction with the gills surface, can be deposited on the gill mucus and gill epithelium or enter the mouth and then the digestive system of mollusks. But not every particle captured by the gills is ingested, as the mussels’ selectivity allows them to sort out inedible or large particles like pseudofeces. However, particles that are smaller in size than their normal food are freely ingested and more easily digested by the mollusks [39,46]. Early studies have already shown the ability of mussels to absorb polystyrene microspheres ranging in size from 3 to 10 µm and transport them into the circulatory system [13,47]. Penetration of microplastics of different sizes into organs and tissues of mollusks favors their active accumulation in the gut. Microspheres of different polymers, ranging in size from 0.1 to 90 μm, have been shown to concentrate within hours in the digestive organs and other tissues of *Mytilus* sp. and to remain there for a long time [39]. Larger particles (>20 µm) are removed from the body first, whereas smaller particles are still present in the gut and hemolymph after their three-day purification and can migrate into the bloodstream, persisting there for up to 48 days [39,48]. Subsequent uptake of the polymeric microspheres by the surface of the gastrointestinal tract occurs by endocytosis and by granulocytes. Then, the microspheres are transported to lysosomes, hemolymph, and other organs [39,40,49,50]. This indicates the direct contact of polymer microparticles with cell organelles.

According to these studies, the 0.9 µm µPS used in our work could actively settle on the gills, penetrate the digestive tract, and hemolymph, affecting each tissue directly. Intensive accumulation of lipid oxidation products and decreased antioxidant activity in the cells of the organs studied also indicated the toxic effect of µPS and the development of oxidative stress in the mollusk organism. It was shown that even short-term exposures of polymers’ microparticles to the hydrobionts organs and tissues cells resulted in the formation of oxyradicals and stress proteins, changes in the activity of anti-radical enzymes and the anti-radical system, the accumulation of lipid oxidation products, the destabilization of lysosome membranes, DNA molecule damage, and histological changes and inflammatory reactions with the formation of granulocytes [13,41,51]. At the cellular level, this is shown by the disruption of the DNA molecule structure, the destabilization of lysosomal membranes, and lysosomal compartmentalization, with the effects increasing with chronic exposure [27,47,52]. In mussel gill and digestive gland cells exposed to µPS, we observed the highest rates of DNA damage compared to exposure to HDPE fragments. Comet assay and LMS determination allowed us to efficiently assess short-term exposure to microplastics, and this proved to be one of the sensitive methods in our experiment. Compared to HDPE fragments, the bioavailability of which is related only to washed out additives, µPS possess several mechanisms of effect on the organism that act at once—the release of their own toxic compounds, mechanical damage during their penetration into mollusk organs and tissues, and the generation of oxyradicals.

## 5. Conclusions

Despite the toxicity of both types of polymers, the bioavailability of microplastics to filter-feeding mollusks is higher and more dangerous compared to mesoplastics. The investigated polymers cause a serious threat to the biological system of filter feeding mollusks, irrespective of size and species. The toxic effects of polymers are shown at the genetic and biochemical level; therefore, analyzing the effects of different types of plastics from macro- to micro-size is important in environmental and organismal risk assessment.

## Data Availability

Dataset available on request from the authors.
